# Considerations for Pediatric Retirement from Athletics Following Repetitive Concussive Traumatic Brain Injury: Incorporating the Right to an Open Future

**DOI:** 10.3390/ijerph18052266

**Published:** 2021-02-25

**Authors:** Tyler S. Gibb, Kathryn Redinger, Casey Fealko, Sonia Parikh

**Affiliations:** Western Michigan University Homer Stryker M.D. School of Medicine, Kalamazoo, MI 49008, USA; Kathryn.Redinger@med.wmich.edu (K.R.); Casey.Fealko@med.wmich.edu (C.F.); Sonia.Parikh@med.wmich.edu (S.P.)

**Keywords:** pediatric retirement, concussion, sports-related injury, ethical framework

## Abstract

Guidance regarding the decision to remove an adolescent from athletic competition immediately following an acute concussive injury and the safe return of play in the short term is widely accepted and supported by clinical evidence, local institutional policies, and state and federal laws. There is considerably less guidance regarding the decision to permanently retire an adolescent athlete for medical reasons due to concussive injuries. In this article, we discuss the clinical and non-clinical considerations that should guide clinicians in discussions regarding the adolescent athlete’s permanent retirement by emphasizing the ethical obligation to protect the child’s right to an open future as possibly determinative in otherwise ambiguous cases.

## 1. Introduction

Parents, pediatric athletes, school sports leaders, and health care providers are more attentive than ever about the risks and long-term impact of concussive injuries due to athletic competition. As many as 1.1 million to 1.9 million pediatric recreational and sports-related concussions are estimated to occur annually in the U.S. [1]. According to the 2016 Berlin expert panel, a sports-related concussion (SRC) is a “traumatic brain injury induced by biomechanical forces” [2]. It may be caused by a “direct blow to the head, face, neck or elsewhere on the body with an impulsive force transmitted to the head” and typically results in “the rapid onset of short-lived impairment of neurological function that resolves spontaneously” [2]. Concussions may include a constellation of clinical neurologic, cognitive, and emotional symptoms. Although widely prevalent, concussions present unique diagnostic and treatment challenges compared to other disease processes, mainly due to its seemingly invisible nature, including a lack of objective findings on physical examination, laboratory studies, and imaging. This ambiguous disease process, however, is not benign. Time to recovery and the reduction of long-term symptoms and complications in the pediatric athlete’s developing brain is therefore dependent on early diagnostic recognition of symptoms [3].

Research, public health campaigns, and legislation have focused on educating concussion symptoms awareness, the need for removal from play, and effective management strategies, including the decision to return to play in the acute injury stage. However, despite these crucial efforts, empirical evidence demonstrating long-term success is still lacking [4]. The decision to medically retire an athlete can be complicated, emotionally charged, and should be based on much more than merely the number of concussions the athlete has sustained [5,6,7,8,9]. The decision can be even more problematic when the athlete is an adolescent. For pediatric patients who have suffered a concussion while playing sports, the idea of retiring from sports may seem drastic and heavy handed. Facing a future without sports may even challenge the adolescent’s emerging personal identity and social connections. The factors relevant to the medical retirement decisions are numerous, and the risks and benefits of each must be weighed in light of the individual player’s circumstances. Much of the literature published on this topic, and many others in clinical ethics, reduces these deliberations to mere benefit and harm balancing [9]. In this paper, we suggest framing the problematic decision of a pediatric athlete’s medical retirement from participation in sports due to concussive injuries in relation to the adolescent’s right to an open future [10,11]. An adolescent’s right to an open future is an ethical construct, first articulated by Feinberg, that aims to “protect the child against having important life choices determined by others before she has the ability to make them for herself” [11]. As Davis-Hayes et al. detailed, although not specifically in the context of pediatric patients, many clinical and non-clinical factors must be balanced when considering medical retirement. There are times when a detailed balancing of relevant factors fails to yield a clear path forward. We argue that assessing a child’s right to an open future may prove useful in some ambiguous cases.

## 2. Recognition of Duty and Conflicts of Interest

The issue of concussions may generate considerable ethical tensions for team physicians, who may feel competing duties to patients, teams, coaches, and employers. However, the physician’s first duty is to her patient’s health and well-being [12]. The physician’s duty to safeguard the pediatric athlete’s present well-being is well established, but we emphasize the duty to protect the child’s future well-being. One formulation of that obligation is that a child has a right to exercise all her autonomous rights at some point in the future. To respect this right, her caregivers (including parents, coaches, guardians, teachers, etc.) have an ethical duty to optimize, in so far as is reasonable and influence may be brought to bear, the “actions and objects… [that] could make a substantial difference to the nature and quality of the child’s life” [11]. Restated, caregivers must attempt, to the best of their ability, to facilitate their opportunity to develop capacities, skills, preferences, and options for exercising autonomy in the future. Feinberg and Millum carefully develop the character, foundation, and limits of this ethical duty in great philosophical detail elsewhere, which is outside the scope of this article [10,11].

One first step towards discharging this professional and ethical duty is to optimize the quality of information upon which decisions will be based. Precise diagnostic criteria, treatment plans, and data about prognosis are notoriously elusive for SRC patients, but more so in the pediatric context. The pediatric healthcare provider must take a family-centered approach to her discussion and counseling regarding medical retirement due to the numerous relevant stakeholders.

A challenge to SRC management, in particular, is that symptoms are generally subjective and must be self-reported. A physician must be aware of outside pressures on players to underreport symptoms and counsel appropriately about the potential dangers of worse symptoms and even catastrophic neurologic consequences of second impact syndrome if they are to be injured while their brain is still vulnerable [13]. Previous studies have suggested that conflicts of interest between doctors, patients, and teams may substantially hinder the proper adherence to concussion guidelines [14]. However, in a survey published in 2015, Kroshus et al. found that among 328 male and female collegiate athletes, even when living away from home, athletes perceived pressure from a parent or guardian to continue to play after an injury more than they perceived pressure from coaching staff [13].

## 3. Clinical Factors

When assessing whether a pediatric athlete should be retired, physicians must give clinical factors significant considerations. The clinical contraindications of continuing sport participation may supersede all other aspects in some cases. The cumulative impact of multiple concussions, regardless of whether the history is of minor or severe concussions, is significant and relatively understandable for many stakeholders in these decisions. “The greatest predictor of a recurrent concussion is [a] history of concussion” [15]. Although there is no specific number of concussions that indicates when a pediatric patient should retire from the sport, multiple past head injuries and the correlating recovery history can provide a relatively more objective indicator of risk.

The number of concussions, while important, is not the sole indicator for potential retirement [16]. The severity of past concussive injuries should also be considered. Several minor concussive injuries are as relevant as serious concussions with loss of consciousness, although some stakeholders may not fully understand that fact. The decreased threshold to injury and progressively longer recovery times after subsequent injuries are prudent indications to recommend retirement [15].

The timing and frequency of concussions are essential to consider as well. It is widely accepted that an athlete should not return to play after a concussion while still suffering symptoms of the injury [15]. Although there is a lack of empirical evidence to confirm this, it stands to reason that repetitive concussive injuries sustained in a short space of time, such as in the same season, may portend a worse long-term prognosis than similar repetitive injuries over several years or seasons.

There is a risk of post-concussion syndrome (PCS) with any concussion. PCS, also known as a neurocognitive disorder due to brain injury, is a complex disorder in which symptoms from a concussion may persist for weeks or months [15]. PCS is relatively common, with 1 in 7 concussion patients suffering from persistent problems post-concussion [15]. One Canadian multi-institutional group recommends an athlete with prolonged PCS (>3–6 months) should be strongly encouraged to retire for the season [4]. In a 2017 metanalysis reviewing pediatric SRCs from 18 studies, the recurring predictor variables of the prolonged post-concussive syndrome were acute headache, migraine, and dizziness (all when higher than preinjury levels), as well as female sex and history of receiving multiple concussions [17].

The effects of SRC in children are different from adults. Children’s brains are more susceptible to long-term damage from a concussion than the brains of adults [18,19]. Physicians should be aware that clinical concussion guidelines are largely derived from high school and collegiate athletes’ data. In a meta-analysis published in 2017, Davis et al. reveal that the currently available literature does not adequately address which age groups in which children with SRC should be managed differently from adults [17]. Additionally, there is no consistent evidence to indicate optimal timing for children or adolescents to return to sports play compared to adults [20].

In their 2018 guidelines for medical retirement from sports after a concussion, Davis-Hayes et al. describe specific clinical characteristics as absolute and relative contraindications to RTS [9]. Absolute contraindications include evidence of structural brain injury identified on neuroimaging. Results of conventional neuroimaging are typically normal in sports-related concussive injury, and while they may be indicated to rule out more serious traumatic brain injury or other pathology, in general, they offer little utility to the diagnostic or treatment decisions of the pediatric athlete. Additionally, CT exposes children to the potentially harmful effects of ionizing radiation, which increases the risk for benign and malignant neoplasms. Clinical prediction rules to identify pediatric patients with a very low risk of clinically important traumatic brain injury (ciTBI) following blunt head trauma. Clinicians could avoid neuroimaging and are becoming better established and validated [21].

If data from advanced imaging is available, it should be considered when deciding on medical retirement. Some organizations suggest sport retirement for “any athlete with a traumatic abnormality detected on neuroimaging” due to the potential risk of further injury [4]. When non-traumatic structural abnormalities are identified, the relative risk and contraindications for each specific abnormality must be considered [4]. For example, a cavum septum pellucidum poses no relative contraindications to returning to play. In contrast, a Chiari Type 1 malformation poses a low but potential risk of irreversible injury or death after even mild head trauma [22].

Relative contraindications include (1) post-concussive signs lasting more than 90 days or increasing in severity, (2) cognitive impairment (as demonstrated on neuropsychological testing), (3) diminished academic performance or social engagement, and (4) decreased concussion threshold or decreased interval between concussions [9]. While the authors say that any one of these may be managed and does not necessitate retirement, the totality of clinical factors present may lead to the clinical recommendation for medical retirement.

Biological sex seems to be associated with different risks of concussions. Females are more likely to suffer concussions than males, and there also appears to be a difference in outcomes for traumatic brain injuries and concussions between males and females [23]. However, these observations may also be due to differences in self-reporting of symptoms between males and females. Females athletes tend to report symptoms of a concussion more often and are more likely to be diagnosed with concussions than male athletes [15]. Thus, it is unclear whether an athlete’s sex plays an actual physiological role in the risk for concussions. However, evidence suggests females take longer to recover from concussions than males, although it is not known if this is due to physiological differences or other factors [24].

If an athlete or parent does not understand the signs and symptoms, they may not quickly recognize them, leading to increased damage. At the time of injury, an athlete may not even realize they have suffered a concussion, making it even more critical for people surrounding the athlete to be trained to recognize these injuries. Even with proper education, vague symptoms may skew parents’ perceptions of their child and their health. Whether or not an athlete can appreciate a concussion’s risks is essential, especially the dangers of playing with a concussion. Athletes who delay reporting concussion symptoms and continue to play prolong their recovery [15]. The presence of an athletic trainer does not necessarily mitigate this risk. A 2016 study of high school athletes showed that those with access to an athletic trainer had more concussion knowledge but did not report suspected concussions more often than those without [25].

Current evidence suggests children who participate in contact and collision sports are not at risk for long-term neurologic or psychiatric consequences [15]. However, the risks of head trauma are evident. In 2014, a study on the Swedish population showed that a history of at least one traumatic brain injury (TBI) was associated with a significantly increased risk for premature mortality, particularly from suicide, injuries, and assaults [26]. Additionally, increased rates of psychiatric disorders were associated with a history of at least one TBI, particularly for substance use disorders and depression. This study was not specific to children nor sports-related concussions. Nevertheless, it highlights the potential long-term neurologic and psychiatric consequences of traumatic brain injuries [26].

Another neurologic consequence of repeated head trauma gaining further coverage and research since its discovery in 2002 is Chronic Traumatic Encephalopathy (CTE). CTE is a progressive neurodegenerative disease associated with repeated trauma to the head [27]. The trauma associated with CTE includes concussions as well as subconcussive hits. CTE results in memory loss, confusion, aggression, depression, suicidality, and progressive dementia. Currently, CTE can only be diagnosed at autopsy after a person dies. There is no cure for CTE, and the only way to prevent it is to avoid repeated head trauma. Most CTE cases are reported in collegiate and professional athletes whose brains had been exposed to trauma, typically multiple episodes [4]. Yet, there are rare reported CTE cases in adolescents with a history of repetitive trauma [27].

## 4. Non-Clinical Factors

When deciding the fate of a pediatric athlete’s sports participation, the viewpoints of various stakeholders, including parents, pediatric athletes, school sports leaders, and health care providers, are all critical. Each stakeholder has their own calculation of the relative value of sports participation. These individual calculations include the financial toll of sports participation, which may be significant, including purchasing necessary equipment, travel expenses, coaching fees and lessons, and registration costs. The potential health and social benefits of sport participation are also relevant to the continued involvement. While injuries and hard hits are common in some sports, parents have varying opinions regarding the lasting effects of such injuries relative to social, financial, and other factors beyond mere health considerations.

The type of sport and level of competition at which it is played will impact the risk of concussions. Merely understanding a parent’s preferences is also insufficient. One must also consider the goals, aspirations, and feelings of the pediatric athlete. Participating in organized sports allows children to form friendships, camaraderie, and further develop physical and social skills and deepen their love for the sport.

These benefits should be accounted for when deciding whether a child should retire from one particular sport, but, perhaps, not from all sports. Combat sports, such as boxing, kickboxing, and MMA, have the highest risk of concussions, followed by collision, contact, and noncontact sports [4]. Similarly, the level of competition is essential to examine. Some sports already have mechanisms in place to reduce trauma to the head in younger athletes. USA Hockey does not allow body checking until after the age of 12 [28] because there is some evidence that limiting body checking in youth hockey reduces the risk of concussion [4].

Similarly, the American Youth Soccer Organization (AYSO) does not allow players to head the soccer ball until after age 12 [29]. Participation in youth tackle football has generated much discussion, with calls for health professionals to take a stance in opposition of the sport as a whole due to the burden of pediatric SRC attributed to youth tackle football alone. Football is the leading cause of sports-related injuries, twice that of the second most popular sport, baseball [30]. Although the frequency of concussions in football is about the same as in hockey, 50 times as many students play football than hockey, football causes far more brain injuries [31]. Football players who sustain an SRC are three times more likely to suffer a second concussion within the same season than non-injured players [32].

Playing sports allows children to remain physically active and practice healthy exercise habits from a young age [33]. Exercise has been proven to improve physical health and plays a preventative role in reducing the risk of future cardiovascular disease [34]. Participation in safe, regular exercise is beneficial to children. The 2018 Physical Activity Guidelines for Americans recommend children and adolescents aged 6–17 should do 60 min or more of moderate-to-vigorous physical activity each day for benefits that include improved bone health, weight, and cognitive function [35]. However, there appears to be a discordance between physical activity guidelines and individuals’ beliefs regarding the benefits of physical activity. In one cross-sectional study of 310 participants, more than half of the respondents reported erroneous beliefs regarding physical activity guidelines [34]. Participating in sports facilitates the development of cardiovascular health as well as gross and fine motor skill development. For some children, organized sports are some of the best, or only, options for regular exercise.

Being a part of a team and playing a sport holds many benefits beyond the physical. Team dynamics allow for strong and healthy relationships between teammates and coaches and may create a support system for the athlete with authority figures and non-parental adults. Participation in sports challenges athletes to develop time-management skills so that they continue to excel in school but are also able to excel in their sport [36]. Sports, organized or informal, may also be one of the few avenues for friend making, social network development, and teamwork.

Many coaches and supporters of organized sports will attest to the benefits of hard work, perseverance, and self-sacrifice required in sports. Developing pro-social characteristics may be the most significant benefit of participation. Removing a pediatric athlete from participation may have a significant mental and emotional toll. Distinguishing mood disorders from post-concussion syndrome can be difficult as athletes may be at risk for mood disorders if they are removed from their activities [37].

Sports represent deep societal and rich community traditions. When deciding to retire or continue participation in a sport, children and parents may focus on potential benefits of sports participation, such as achieving a collegiate scholarship that will finance higher education or a career in professional sports with high earnings potential. This optimistic focus may lead to a tendency to discount the risks associated with youth participation. Children and parents of lower socioeconomic status may have the perspective that the potential of education and future earnings outweighs more immediate physical risks, particularly if excelling in sports is viewed as one of the only viable avenues for achieving those goals. Some athletes and parents may be willing to sacrifice physical health for future financial benefits, and some may even view it as an altruistic duty. For some youth athletes, the goal of participating in a sport at the collegiate or professional level, however unlikely, is beneficial for social, behavioral, and, possibly, educational reasons. Sports may be a vehicle for college attendance through scholarships. In some cases, this financial benefit may be the only viable means of pursuing higher education and long-term social and economic benefits that a college education may provide. For context, there are just over 1 million high school football players. In total, 6.5% go on to play in college, and 1.2% of college players are drafted into the NFL [38]. Those small numbers are consistent across professional sports. Still, they stand in stark contrast to survey data from the study “Sports and Health in America,” conducted by National Public Radio, the Robert Wood Johnson Foundation, and Harvard’s T.H. Chan School of Public Health, which reports 26% of parents of high school athletes to believe their child has a chance at a professional athletic career [39].

*The Atlantic* magazine detailed a narrative of a low-income family from a rural town of Georgia. Here, a single mother raised three boys while working full time [40]. When asked her opinion regarding the sport, she described its many positive effects. Sports kept her three boys out of trouble and out of the streets, thus protecting them from gang activity. It allowed them to form strong bonds with their coach, who acted as their mentor and supported them on the field and in their education. They may be able to attend college by earning sports scholarships. She viewed football as an opportunity for her boys to escape the redundant lifestyle. However, she was not oblivious to the injuries and health concerns football poses. She responded that her boys could be injured in a car accident, in a shooting, in gangs, or any other sport. She said, “If it’s meant to happen, it’s going to happen. We can’t stop it. You can get injured in any sport” [40]. She stood by the idea that fear of injury was not a reason to prevent her boys from playing football. While this may be a popular opinion in her town and other families in similar conditions, it is not the case in many others [40]. Interestingly, white youth athletes from high socioeconomic backgrounds in the Northeast, Midwest, and West have been reportedly shifting away from contact heavy sports like football towards lower contact sports, like baseball [40].

Socioeconomic differences also play a significant role in the recovery of a child after a concussion. The effects of prolonged recovery from concussion can cause hardship not only on the athlete but also on their family. Caring for a child with a TBI can alter normal family dynamics, chiefly if the symptoms persist for weeks or months. Families with less flexibility or financial resources may face more significant financial and emotional strain when caring for a child with these long-term symptoms, especially if they may need to take time off work to care for a child.

## 5. Case Examples

Consider the examples of two hypothetical patients. First, Ben is a 14-year-old football player on the freshman team at his high school. He is not an exceptional player and will likely not see significant playing time, even as a senior. This season, Ben has suffered two moderate concussions during practices. His coaches support him continuing to play football but are cautious. Ben is passionate about football and derives many personal and social benefits from participation on the team. His parents also place a high value on Ben’s continued participation as many family members have excelled in the sport. Being a football player is vital to their family culture.

Second, Ashley is a 17-year-old varsity high school volleyball player in her senior season. She has received multiple collegiate scholarship offers. Over the past three seasons, Ashley has suffered numerous head injuries, consistent with minor concussions without losing consciousness. She has recently been suffering from persistent headaches and concentration problems in school. Her coaches strongly support her continuing to play volleyball in high school and at the collegiate level.

Moreover, Ashley would be the first player from the school to receive a volleyball scholarship. Ashley is an outstanding student and has received multiple academic scholarships as well; thus, athletic scholarships are not the only means of attending college. Her parents are ambivalent about her continuing to play the sport.

Both Ben and Ashley have come to be evaluated by a pediatrician and for recommendations about continuing to participate in their respective sports.

Assessing whether a particular adolescent athlete should be retired from sports due to concussive injuries requires the interrogation and weighing of many clinical and non-clinical factors (See Table 1). The following sections examine the relevant clinical and non-clinical factors that should be considered when making this evaluation but then offer the additional ethical duty to protect the child’s right to an open future as potentially determinative in otherwise ambiguous cases.

## 6. Weighing Risks, Harms, Benefits, and an Open Future

Ben, the 14-year-old football player, and Ashley, the 17-year-old volleyball player, face very different risks and benefits related to continued participation in their sports. Unsurprisingly, in good faith, stakeholders will prioritize and weigh the clinical and non-clinical factors differently in the difficult decision of when an adolescent athlete should be retired. In this section, we revisit the cases introduced above and offer guidance on how stakeholders may navigate these decisions while emphasizing the right to an open future concept. A few ethical principles should guide these decisions—protecting a free and open future, respecting the pediatric athlete’s developing autonomy, parental autonomy, and obligations to optimize holistic benefits while minimizing avoidable harms.

An athletes’ choice to stop participation in a sport should prioritize all other stakeholder’s preferences. In Ashley’s case, regardless of her coaches’ strong preference for her to continue playing volleyball, if she chooses to retire, that should be honored. However, her parents, coaches, and healthcare providers should still explore her decision, but Ashley should not be coerced or manipulated into other stakeholders’ continued participation. Suppose parents continue to pressure an athlete to participate. In that case, we should be concerned for secondary interests (prestige to the sports program or coach, the team or family’s reputational interest, etc.) beyond the benefit of the athlete. Adolescent athletes who have concluded that they do not wish to participate in a sport should be respected as a nascent exercise of their autonomy, which is fundamental to protecting their right to an open future.

A child’s preference to continue to participate in their sport should not carry the same weight as deciding to stop playing. For example, Ben may strongly wish to continue to play football, despite the physical risks. Children are not always in a position to appropriately evaluate future risks and potential harms. Ben and his parents may disproportionately prioritize the social and community benefit of being a football player to his history of concussions’ physical risks. It is possible that a coach, school sports leader, or a health care provider could intervene and unilaterally declare an athlete ineligible to participate. This stance should be a last resort approach, only after discussions with Ben and his family have not resulted in consensus.

Overwhelming physical risk should be considered dispositive when weighed against minimal or even moderate non-clinical benefits. The opposite should also be true—overwhelming non-clinical benefits should prevail over minimal or even moderate clinical risks and harms. Ambiguous situations, such a Ben’s and Ashley’s, are more challenging to navigate and require additional ethical inquiry, which is the normative value of the right to an open future in these discussions. If no clear answer is reached by weighing the clinical and non-clinical risks, harms, and benefits, stakeholders ought to evaluate to the extent to which the adolescent athlete’s open future may be meaningfully curtailed by continued participation. This is not merely an assessment of future benefit but emphasizes the future exercise of the athlete’s autonomy as unencumbered as possible. It may be obvious, from this perspective, how impactful neurological injury is, for example, not just to the physical well-being of the athlete but also in terms of the choices, experiences, and actions that are available to her in the future. Equally challenging is when parents or guardians disagree, particularly if one parent has a strong preference in continuing participation while the other parent strongly opposes—in many situations, having all stakeholders discuss their priorities and which factors they deem to be most important may be helpful reducing intuitive or emotional decision making. The use of decision-making trees can provide families and clinicians a more structured approach to discussing medical retirement [9].

Ben’s case presents serious clinical risks and harms, based on the severity of his injuries and their timing. However, Ben also stands to experience significant non-clinical benefits as well. Other stakeholders, such as healthcare providers and coaches, may discuss establishing criteria for continued participation and indicators when participation should stop. For example, after Ben is non-symptomatic from his most recent injury, an agreement may be reached about retirement after any subsequent concussions. Alternatively, they may agree Ben should sit out the current season but may return in the future. If continuing to be involved in football is important to Ben and his family, other modes of participation should be explored, such as being a student manager or coach’s assistant.

Similarly, Ashley, her parents, and coaches may agree about reducing the extent of her participation—for example, less practice time to facilitate her finishing out her senior season. This would prioritize her open future by reducing short and long-term risk while leaving the widest degree of options available in the future. Creativity and honest communication will be essential in these discussions.

Healthcare providers have an important, but rarely a definitive, role in navigating ambiguous decisions about when to retire an athlete. Ashley’s case also provides a reminder to clinicians that they should consider the diverse sports and activities that pose a risk for concussions, rather than just the prototypical male athlete participating in contact sports. The healthcare provider’s role should be as an advisor of the clinical implications of participation, both the benefits and the risks, and advocate for the non-clinical benefits of sports participation. Except in extreme situations, the prerogative to retire an athlete should rest with the athlete, parents, and coaches. Healthcare providers have an important role in identifying and clarifying the factors critical in making this challenging decision, particularly in how the current clinical information is relevant to discussions of future consequences that may bear on the adolescent athlete’s pursuit of a full and fulfilling life. However, healthcare providers should only attempt to prohibit sports participation when there is little reasonable doubt that the athlete is at significant risk of short or long-term harm, and the other stakeholders are not fully appreciating or taking into consideration that serious risk.

## 7. Conclusions

With the increase in concussion reporting comes the increased concern for the effects of concussions on the developing brain. There are no evidence-based guidelines indicating when a pediatric patient should medically retire from a sport. This paper highlights several important considerations that parents, pediatric athletes, school sports leaders, and health care providers should consider when making the difficult decision regarding medical retirement from sports, but emphasizes the less commonly considered ethical duty to protect a child’s right to an open future. While many organizations have offered recommendations, they are often conflicting or only apply to very discrete situations, most of which do not apply to the general population. In the absence of evidence-based guidelines, the parents, pediatric athletes, school sports leaders, and health care providers should together weigh the risks and benefits to determine whether a pediatric patient should be medically retired, always with an eye towards an open future.

## Figures and Tables

**Table 1:** Factors to consider when to retire a child athlete from sports post-concussive injuries.

Clinical Factors	Non-Clinical Factors
Number and Severity of Past Concussions	Type of Sport and Level of Competition
Temporal Spacing of Concussions	Aspirations of Long-term participation
Post Concussive Syndrome	Physical, Social, Mental & Emotional Benefits
Neuroimaging Findings	Socioeconomic Considerations
Age and Sex	

## Data Availability

No new data were created or analyzed in this study. Data sharing is not applicable to this article.
